# Major Depression Impairs the Use of Reward Values for Decision-Making

**DOI:** 10.1038/s41598-018-31730-w

**Published:** 2018-09-14

**Authors:** Samuel Rupprechter, Aistis Stankevicius, Quentin J. M. Huys, J. Douglas Steele, Peggy Seriès

**Affiliations:** 1 Institute for Adaptive and Neural Computation, University of Edinburgh, Edinburgh, United Kingdom; 2 Centre for Addictive Disorders, Hospital of Psychiatry, University of Zurich, Zurich, Switzerland; 3 Translational Neuromodeling Unit, Institute of Biomedical Engineering, University of Zurich and ETH Zurich, Zurich, Switzerland; 4 Division of Imaging Science and Technology, Medical School, University of Dundee, Dundee, United Kingdom

## Abstract

Depression is a debilitating condition with a high prevalence. Depressed patients have been shown to be diminished in their ability to integrate their reinforcement history to adjust future behaviour during instrumental reward learning tasks. Here, we tested whether such impairments could also be observed in a Pavlovian conditioning task. We recruited and analysed 32 subjects, 15 with depression and 17 healthy controls, to study behavioural group differences in learning and decision-making. Participants had to estimate the probability of some fractal stimuli to be associated with a binary reward, based on a few passive observations. They then had to make a choice between one of the observed fractals and another target for which the reward probability was explicitly given. Computational modelling was used to succinctly describe participants’ behaviour. Patients performed worse than controls at the task. Computational modelling revealed that this was caused by behavioural impairments during both learning and decision phases. Depressed subjects showed lower memory of observed rewards and had an impaired ability to use internal value estimations to guide decision-making in our task.

## Introduction

Although major depressive disorder (MDD) is a debilitating condition with a high prevalence and substantial economic impact^1^. A core symptom of clinical depression is anhedonia^2^ and patients often display impairments in executive function, working memory and attention^3,4^.

Another common symptom during depressive episodes is “bleak and pessimistic views of the future”^2^. The theory of learned helplessness posits that people with a pessimistic explanatory style (attributing their helplessness to a stable, global, internal cause) are at greater risk of developing depression^5^. There exists extensive evidence that patients diagnosed with MDD exhibit features of Beck’s Negative Cognitive Triad, which is characterized by negative and pessimistic views about oneself, the world and the future^6^, consistent with a pervasive pessimistic cognitive bias. The Beck Depression Inventory (BDI^7^) and the Beck Hopelessness Scale (BHS^8^) both measure aspects of this triad and Cognitive Behavioural Therapy (CBT), which targets these negative biases can be an effective treatment for depression^9,10^. Here we used a novel experimental paradigm and computational models of decision-making in order to supplement these subjective clinical interviews and rating scales with objective behavioural evidence.

Behavioural impairment in MDD has consistently been found with at least two tasks (see Chen and colleagues^11^ for a review): the Iowa Gambling Task (see Must and colleagues^12^ for a mini review) and the Signal Detection Task (see Huys and colleagues^13^ for a meta-analysis). In both paradigms, participants repeatedly choose between options and observe probabilistic reward outcomes based on their choices. Depressed patients are impaired in their ability to properly integrate their reinforcement history to adjust future behaviour.

We used a probabilistic reward-learning task, which has previously been reported to demonstrate individual behavioural differences that were associated with Life Orientation Test — Revised (LOT-R; measuring optimism) scores^14^, as well as neuroticism scores (see Supplement) in healthy subjects. In the task, participants were asked to maximize their rewards by choosing between fractal stimuli, for which they could estimate the probability of reward from previous passive observations, and another target associated with an explicit reward probability value. Here we tested patients with depression as well as healthy controls and used a computational modelling approach to describe their behaviour. This allowed us to formulate specific hypotheses, corresponding to distinct computational models, about both the learning and the decision process during the task. While focusing on group differences, we also explored how participants’ ratings of depression severity, optimism and neuroticism affected their performance across groups.

Specifically, we tested whether there was objective evidence for: (a) a behavioural difference in learning and decision-making between MDD subjects and healthy controls, and (b) a pessimistic bias about the likelihood of reward in MDD, and then performed exploratory analyses, probing for (c) a correlation between computational model parameters and ratings of depression severity or neuroticism.

## Methods and Materials

### Participants

The main dataset analysed here consists of thirty-nine subjects (Tables 1 and S1) including 19 patients meeting DSM-IV criteria for a diagnosis of MDD and 20 control participants without a history of depression or other psychiatric disorder. The task was performed during fMRI scanning and in the following this will be referred to as “fMRI dataset”. Importantly, patients were unmedicated. Diagnosis was made according to the MINI PLUS (v5.0) structured diagnostic interview^15^. The mean BDI score of the patient group (24.7) can be regarded as “moderate severity” depression (see Supplement for additional information on questionnaire scores). Data collection took place at the Clinical Research Imaging Centre, Ninewells Hospital and Medical School, Dundee. The study was approved by the East of Scotland Research Ethics Service (UK Research Ethics Committee, study reference 13/ES/0043) and all experiments were performed in accordance with relevant guidelines and regulations. Written informed consent was obtained from all subjects.

**Table 1:** Demographics of participants from both dataset versions (see Table S1 for more details). BDI, Beck Depression Inventory; LOTR, Life Orientation Test – Revised; NART, National Adult Reading Test; Data given as n or mean ± std. Due to the small number of Pilot patients, standard deviations are not shown for this group.

Group	No. Subjects	Age	Sex (F/M)	BDI	Neuroticism	LOT-R	NART
fMRI Patients	15	17–41	12/3	24.7 ± 13.1	46.3 ± 7.1	9.1 ± 5.5	46.8 ± 4.2
fMRI Controls	17	18–33	13/4	4.2 ± 5.6	29.8 ± 8.0	18.4 ± 3.1	46.6 ± 3.2
Pilot Patients	3	N/A	N/A	27.7	50.7	9.3	45.3
Pilot Controls	21	N/A	N/A	10.1 ± 12.2	34.4 ± 11.5	14.5 ± 5.5	44.0 ± 11.3

MDD and control groups of the fMRI dataset were matched for age, sex and National Adult Reading Test (NART) scores, which were used to estimate premorbid IQ^16^. Exclusion criteria included claustrophobia, serious physical illness, pre-existing cerebrovascular, neurological disease, previous history of significant head injury, and receipt of any medication likely to affect brain function. All subjects were recruited using the University of Dundee advertisement system HERMES and were paid £20 plus up to £10 dependent on task performance. Four patients and three controls were excluded from further analysis from the fMRI dataset, after performance results showed that they did not choose the higher reward (in the 48 trials in which the reward probability was not the same) in at least 50% of cases. Two additional participants were excluded from all analysis, because they did not complete the study. Model comparison and primary data analysis, which used the fMRI dataset, therefore included 15 participants with MDD and 17 controls.

To further validate our results, we also analysed a second dataset we had previously collected to validate the experiment outside the scanner. In the following, this will be referred to as “Pilot dataset”. It included 3 MDD and 21 control participants (Tables 1 and S1). Recruitment and assessment was performed in the same way as above and the same ethics statement applies. Model comparison was performed on the fMRI dataset and the best performing model was then separately fitted to the Pilot dataset.

### Experiment

**Figure 1:**
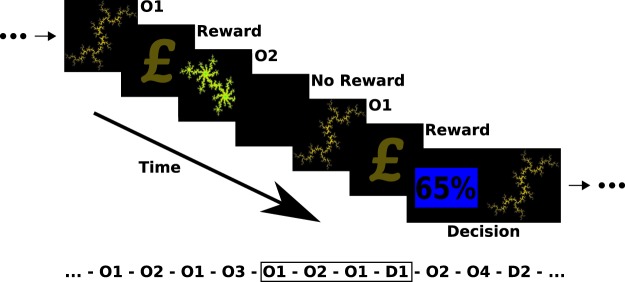
Experimental paradigm. Subject passively observed different fractal stimuli which were followed by reward (a pound symbol) or no reward (blank screen). Interleaved with these observations were decision prompts in which they had to make a choice between one of the observed fractals (for which they could estimate reward probability) and an explicit numeric probability value in order to maximize their reward. An example of a longer sequence is shown at the bottom with the encased subsequence depicted above.

The paradigm (Fig. 1) was adapted from Stankevicius and colleagues^14^. The experiment was implemented in MATLAB R2007b (The MathWorks, Inc., Natick, MA) using the Psychophysics Toolbox^17–19^. Additional details about the experiment are provided in the Supplement and the fMRI analysis will be reported elsewhere. Here we focus on behavioural differences, model fitting and best model identification.

Participants passively observed fractal stimuli, which were followed by either a reward (depicted by a pound symbol) or no reward (no symbol). Interleaved with these observations were decision screens, during which they were asked to make a choice between one of the fractal stimuli they had observed, and an explicit numeric probability value. Participants were asked to choose the higher probability (or reward) value option, which required them to estimate the value of the fractal stimuli they had observed. There were seven possible differences in the numeric value probability. Either option could have a higher probability value of 10%, 20% or 30% (each of which was the case for 8 decision trials) or they could have the same probability of reward (in 12 trials). Our Pilot dataset used a slightly different task, in which possible differences ranged from −90% to +90% instead (in 10% intervals, each displayed in 4 decision trials).

Participants observed a variable number of fractals between decision screens, but each fractal was observed exactly four times before it was used within a decision. Each fractal was used in a single decision and in total participants made 60 decisions (and therefore observed fractals 240 times). The sequence of observations and decisions was pseudo-random, and identical for all subjects. Performance feedback was only given at the end of the experiment. Data collection for each subject lasted approximately 2 hours, which included collection of rating scale data (see Table S1).

### Behavioural Performance Data Analysis

We tested for differences in average reaction time, IQ and other questionnaire scores between the groups using Welch’s t-tests. We measured participants’ performance in terms of how often the fractal was chosen as a function of the difference between the probabilities of the two options (assuming exact estimations for the fractal probabilities; i.e. if a fractal was followed by reward three times, and followed by non-reward once, the fractal probability would be 75%). We fitted a sigmoid function with two parameters (intercept *α*, slope *β*) to the psychometric curves of individuals:

1 $$\varsigma (x)=\alpha +\frac{1}{1+\exp (\,-\,\beta \times x)}.$$

### Computational Modelling

Three different families of models were fitted to the data (see Table 2 for a summary), representing distinct hypotheses about how participants make decisions during the task. All models assume that participants estimate an internal “value” for each fractal they observe and compare this value to the displayed probability when asked to make a choice.

**Table 2:** Model specification. The second column shows how internal values for a fractal *i* are updated after observing an outcome *r* in trial *t*. The *Bayesian* model does not model learning on a trial-by-trial basis. The third column depicts the choice rule that is used to calculate the probability of choosing the fractal over the alternative option (Equation 3). The initial value is set to zero or modelled by *v*_0_. *ϕ*_*i*_ is the displayed probability when asked to make a choice for fractal *i*. *ε* is the learning rate; *β* is the inverse temperature parameter; *A* is the memory parameter; *ρ* is the reward sensitivity parameter; *α* and *γ* are the parameters of the Beta prior. *N*_*i*_ and *n*_*i*_ are the number of times a fractal *i* was observed and followed by reward respectively. See main text and Supplement for additional details.

Name	V update	p(choose fractal i)	Parameters
RL-basic	${V}_{i}^{t+1}={V}_{i}^{t}+\varepsilon ({r}_{i}^{t}-{V}_{i}^{t})$	$\sigma (\beta ({V}_{i}^{t}-{\varphi }_{i}))$	*v*_0_, $\varepsilon$, $\beta$
RL-learning	${V}_{i}^{t+1}={V}_{i}^{t}+{\varepsilon }^{+}(1-{V}_{i}^{t}){r}_{i}^{t}+{\varepsilon }^{-}{V}_{i}^{t}(1-{r}_{i}^{t})$	$\sigma (\beta ({V}_{i}^{t}-{\varphi }_{i}))$	*v*_0_, *ε*^+^, *ε*^−^, *β*
RL-unbiased	${V}_{i}^{t+1}={V}_{i}^{t}+\varepsilon ({r}_{i}^{t}-{V}_{i}^{t})$	$\sigma (\beta ({V}_{i}^{t}-{\varphi }_{i}))$	*ε*, *β*
RL-learning-unbiased	${V}_{i}^{t+1}={V}_{i}^{t}+{\varepsilon }^{+}(1-{V}_{i}^{t}){r}_{i}^{t}+{\varepsilon }^{-}{V}_{i}^{t}(1-{r}_{i}^{t})$	$\sigma (\beta ({V}_{i}^{t}-{\varphi }_{i}))$	*ε*^+^, *ε*^−^, *β*
Leaky	${V}_{i}^{t+1}=A{V}_{i}^{t}+{r}_{i}^{t}$	$\sigma (\beta ({V}_{i}^{t}/4-{\varphi }_{i}))$	*A*, *β*
Leaky-*ρ*	${V}_{i}^{t+1}=A{V}_{i}^{t}+\rho {r}_{i}^{t}$	$\sigma (\beta ({V}_{i}^{t}\mathrm{/4}-{\varphi }_{i}))$	*A*, *ρ*, *β*
Bayesian	${V}_{i}=\frac{{n}_{i}+\alpha }{{N}_{i}+\alpha +\gamma }$	$\sigma (\beta ({V}_{i}-{\varphi }_{i}))$	*α*, *β*, *γ*

First, we fitted variations of a family of reinforcement learning (RL) models that incorporate trial-by-trial prediction errors and a learning rate parameter. During each trial, the fractal is associated with an expectation about reward based on the internal value and this expectation is updated after observing the reward or lack thereof. Such RL models have been used extensively to describe reward-based learning and much research has gone into understanding the connection between prediction errors and the dopamine system^20^. In two of the models (‘RL-basic’ and ‘RL-learning’), the initial value parameter was allowed to vary between 0 and 1, and could therefore act in a similar way as the mean of the prior belief in the Bayesian model (see below). The other two RL models (‘RL-unbiased’ and ‘RL-learning-unbiased’) kept the bias parameter fixed at 0.5, which corresponded to a prior belief that reward was equally likely from the fractal or the explicit option. Two of these models (‘RL-learning’ and ‘RL-learning-unbiased’) aimed at testing whether learning was different following rewards versus no-rewards (“punishment”) by including separate learning parameters for each outcome. It has been proposed that there may be heightened asymmetry between learning from positive and negative outcomes in depression and separate learning rate parameters can be used to account for this (see Chen *et al*.^11^ for a review). They were also used in the previous version of this task^14^.

Next, we fitted the winning model of Stankevicius and colleagues^14^ (see Table 2 and Supplement), which tests the hypothesis that subjects behave as Bayesian observers during the task. This model assumed that at the decision time for a given fractal, participants estimate the number of times the fractal was followed by a reward (the likelihood) and combine this evidence with a prior belief about the probability of rewards associated with the fractals. Although the observations are not modelled on a trial-by-trial basis, this model assumes that the likelihood is computed by (implicitly) counting, and perfectly remembering, the number of times each fractal is associated with reward. In the original experiment, Stankevicius and colleagues^14^ found that the mean of the participants’ prior belief distribution correlated positively with their optimism scores (LOT-R). A more recent analysis of the same data also revealed a negative correlation of the prior mean with neuroticism scores (see Supplement). This means optimists and people scoring low on neuroticism overestimated the reward associated with fractal stimuli and that in this task, optimism and neuroticism acted as a prior belief, biasing performance in situations of uncertainty.

This Bayesian model comes with some limitations. First of all, it does not allow us to distinguish between observation and decision phases, because it ignores individual observation trials. More importantly, the model assumes perfect memory of observations, which is an unrealistic assumption, especially since memory impairments in MDD are exceedingly common^3,4,21–23^.

To overcome these limitations, we therefore also fitted two additional trial-by-trial models (‘Leaky’ and ‘Leaky-*ρ*’), which include neither a learning rate nor a prediction error, but which include a discounting factor (also termed a ‘memory’ parameter). Note that the *Leaky* model is equivalent to the Bayesian model assuming a flat prior and non-optimal (“leaky”) memory. Internal value estimates are updated after observing fractal *i* and associated reward *r* at observation *t* as

2 $${V}_{i}^{t+1}=A\times {V}_{i}^{t}+{r}_{i}^{t},$$

where A ($0 < A < 1$) is the memory parameter (the closer it is to 0, the more a subject “forgets” about their observations and the less they take into account previously observed rewards) and ${r}_{i}^{t}=+\,1$ if observation *t* of fractal *i* was rewarded and 0 otherwise. Initial internal values were set to zero. A second model in this family (Leaky-*ρ*) includes a scaling (“reward sensitivity”) parameter on observed rewards, to capture participants’ subjective valuations of observed rewards. Notably, reward processing (dysfunction) has been identified as a promising phenotype of depression^1^.

The probability of choosing an action was calculated by passing estimated and explicitly displayed reward probability values through a softmax function. For the *Leaky* model, fractal *i* was chosen (as opposed to the displayed reward probability *ϕ*_*i*_) with probability

3 $$p({\rm{choose}}\,{\rm{fractal}}\,{\rm{i}})=\sigma (\beta \times (f({V}_{i})-{\varphi }_{i}))=\frac{1}{1+\exp (\,-\,\beta \times (f({V}_{i})-{\varphi }_{i}))},$$

where *f*(*x*) = *x*/4 is a deterministic function which transforms the internal value estimates to a probability comparable to *ϕ*. The shape of the sigmoid function was determined by the *β* parameter. The higher this inverse temperature parameter, the more deterministic decisions become, while lower values lead to “noisier” decision-making. When the values of actions are unknown, this parameter governs the balancing of exploration and exploitation in reinforcement learning^24^. Higher values mean actions are chosen more greedily, lower values lead to suboptimal actions being chosen more often to explore the environment. Here participants were asked to maximize their reward, which means they were asked to always choose the option with the higher probability of reward and there was no advantage of “exploring” the other option. Each fractal was only associated with a single decision and feedback was only given at the end of the experiment and not after each decision. This makes it unlikely that individuals consciously decided to choose the option they thought had a lower probability just to explore the alternative. More plausibly, participants made wrong choices when they either were not certain about what they had observed or had incorrectly estimated the probability of a certain fractal leading to reward. Note that variations in the two parameters (*A* and *β*) produce separable behavioural effects. Beta affects the probability of choosing the option estimated to have higher probability of reward on all decision-trials. Memory primarily affects the trials in which the fractal should have a higher chance of reward (if perfectly estimated) than the displayed numeric probability (see Supplement).

### Model Fitting and Model Comparison

We used model fitting and comparison procedures previously described by Huys and colleagues^25^. Parameters were maximum a posteriori (MAP) estimates incorporating an empirical prior, estimated from the data. Parameters were initialized with maximum likelihood values; then an expectation-maximization procedure was used to iteratively update the estimates (see Supplement). We calculated the integrated Bayesian Information Criterion (iBIC^25^) for all fitted models to find the model that best fitted the data, taking into account complexity. Simulations were run to verify that both the fitting and comparison procedure recovered reasonable parameters and chose the correct type of model when generating and re-fitting data using known parameters and models (see Supplement).

## Results

### Model-free Analysis

A summary of all questionnaire scores of the two groups is displayed in Table S1. National Adult Reading Test (NART) scores indicated no difference in IQ between the groups (*t*(26.3) = 0.158, *p* = 0.876). Overall, participants did not respond in 17 of 1920 trials (0.89%). Mean response times were not significantly different between groups (RT patients *μ* ± *σ* = 2286 ± 455 *ms*; RT controls *μ* ± *σ* = 2185 ± 360 *ms*; *t*(26.6) = 0.692, *p* = 0.495).

**Figure 2:**
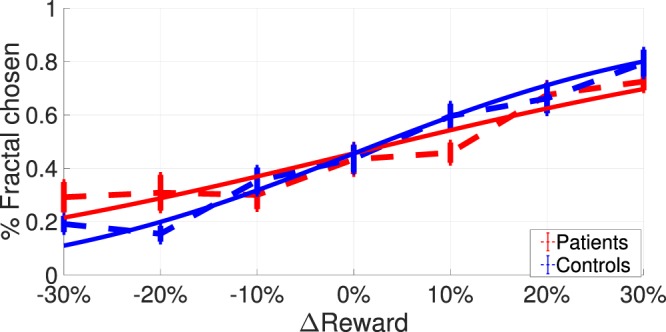
Average sigmoid functions (solid lines) fitted to psychometric curves (dashed lines) of the two groups of the fMRI dataset. Dashed lines depict the average proportion of responses in which the fractal was chosen as a function of the difference between estimated and explicit reward probabilities. Solid lines show the average of simple sigmoid functions fitted to the psychometric curves of individuals. A perfect observer would never choose the fractal when the explicit probability is higher (−30%, −20%, −10%) and always choose the fractal when the estimated probability is higher (10%, 20%, 30%). An unbiased observer would be expected to choose the fractal in half of the trials when reward probability is the same for both options. Error bars represent between subjects standard errors.

Figure 2 shows the fitted sigmoid curves using the average of the fitted parameters for each group. The fitted offset parameter (*α*) was not significantly different between groups (*t*(28.1) = 0.023, *p* = 0.982), but the slope parameter (*β*) was significantly different (*t*(26.3) = −2.383, *p* = 0.025), with controls having steeper curves (*β* controls *μ* ± *σ* = 0.566 ± 0.316), indicating they were significantly better at learning (*β* patients *μ* ± *σ* = 0.350 ± 0.185). Stankevicius and colleagues^14^ recorded a systematic bias in optimistic people towards choosing fractals. We did not find such a systematic bias in healthy participants (as compared to MDD patients) towards choosing fractals, but the difference in the slope parameters indicated performance differences between the groups that we further examined using computational modelling. We were particularly interested in understanding whether those differences stemmed from observation phase or decision phase abnormalities.

### Model-based Analysis

**Figure 3:**
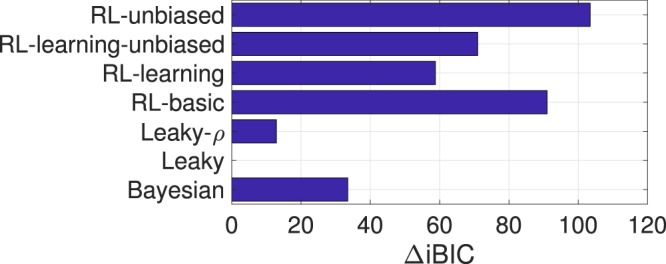
Results of the model comparison. iBIC values of different models relative to the best fitting model *Leaky*. A difference of 10 or higher is considered strong evidence for the model with the lower value^13^.

Model selection using iBIC showed that the *Leaky* model best described participants’ performance in our data (Fig. 3), indicating that in our dataset participants did not seem to rely on their prior beliefs, but were limited by their working memory.

The memory parameter differed significantly between groups (*z* = −2.153, *p* = 0.031; A patients *μ* ± *σ* = 0.90 ± 0.04, median = 0.91; A controls *μ* ± *σ* = 0.92 ± 0.09, median = 0.96). This indicates that patients discounted their estimated values more than controls on each trial, possibly indicating impairments in working memory. The choice sensitivity parameter (*β*) was also significantly different between groups (*z* = −2.341, *p* = 0.019; *β* patients *μ* ± *σ* = 4.67 ± 1.45, *β* controls *μ* ± *σ* = 5.89 ± 1.33), meaning that controls found it easier to follow their internal estimations, while patients chose more randomly. There was a trend suggesting a correlation between parameter estimates (*r* = 0.349, *p* = 0.051). We performed additional simulations by systematically varying the parameters to see if parameter recovery of one parameter was systematically influenced by the other parameter and convinced ourselves that parameter correlation did not cause problems during inference (see Supplement).

We were also interested in understanding whether there existed interesting relationships between model parameters and questionnaire scores. This exploratory analysis revealed a negative relationship between beta and neuroticism across groups (see Supplement). As this was indistinguishable from a group level effect, we then combined our fMRI dataset with our Pilot dataset and focused on healthy participants only. Within the pooled control groups, there was also a significant negative relationship between beta and neuroticism (*t*(35) = −2.679, *p* = 0.011) after controlling for dataset version (Figure S6). This means high neuroticism was related to more variable decision-making in controls.

Further analyses details are reported in the Supplement.

## Discussion

Here we used a probabilistic reward-learning task associated with computational modelling to capture behavioural differences between groups of depressed and healthy participants. We found evidence for impairments in MDD subjects during both learning and decision-making. Our results demonstrate a strong association between depression and participants’ inability to make decisions based on their internal value estimations. MDD patients also showed decreased memory of observed rewards throughout the task. We did not find evidence for a systematic pessimistic bias about the likelihood of reward in depressed participants (see Supplement for a discussion).

Depression is characterized by behavioural, emotional and cognitive symptoms^23^. It is well established that MDD patients display cognitive impairments including deficits in executive function, working memory, attention and psychomotor processing speed^3,4^. Behavioural differences in reinforcement learning performance between groups of depressed and healthy participants have been reported previously (see Chen and colleagues^11^ for a review). In the Iowa Gambling Task subjects repeatedly choose from one of four different decks of cards with different reward and punishment contingencies (unknown to the player). High immediate rewards (or losses in an adapted version) are followed by even higher losses (or rewards) at unpredictable points for some decks. Other decks are associated with lower immediate rewards but even lower unpredictable losses. MDD patients typically choose more often from disadvantageous decks, displaying a worsened sensitivity to discriminating reward and punishment (see Must and colleagues^12^ for a mini review). In the Signal Detection Task participants observe in each trial one of two hard to distinguish stimuli for a very short time and are asked to indicate which stimulus they observed. Correct answers are sometimes rewarded, but unbeknownst to subjects, one of the stimuli is rewarded three times as often as the alternative. Whilst healthy people show a bias towards choosing the more frequently rewarded option, MDD patients do not develop this bias (see Huys and colleagues^13^ for a meta-analysis), an effect thought to be related to anhedonia.

In both the Iowa Gambling Task and the Signal Detection Task participants undergo instrumental conditioning, in which chosen actions are reinforced or punished. Subjects learn from their individual choices and the rewards that follow, and will not experience the same reinforcement history, because their rewards depend on their choices. Findings of differences in behaviour or neural activity between groups therefore have to deal with potentially confounding effects of unequal reinforcement histories. Our experiment contains a Pavlovian conditioning phase, during which conditioned stimuli (fractals) are paired with reward and no choices are made. All participants passively observed the exact same sequence of stimuli and these rewards. Participants could not learn from their instrumental choices in our task, because each fractal stimulus was only associated with a single decision and feedback was only displayed at the end of the experiment.

Computational modelling was used to capture the behaviour of participants during the task and formal model comparison to choose the best fitting model, from which we identified the best fitting parameters for each participant. MDD patients performed worse on our task and the model-based analysis showed that this was due to differences in two model parameters. First, patients discounted (or forgot) previous reward history more than comparison subjects, consistent with reported impairments in working memory and attentional deficits^3,4^. Dombrovski and colleagues found suicide attempters (but intriguingly not non-suicidal depressed elderly people) had lower memory parameter values than control participants in a probabilistic reversal learning task^26^. Our finding is also consistent with another recent study by Pulcu and colleagues which reported increased discounting of rewards in MDD^27^, although discounting in our task was related to past rewards, while Pulcu and colleagues’ task involved future rewards. Notably, a link between working memory and delay discounting has previously been reported^28,29^. Second, we found MDD patients had more difficulty following their internal value estimations of different stimuli, making decisions more randomly. It is possible that patients had a lower confidence in their ability to perform the task, similar to how learned helplessness theories view depression as a consequence of an organism’s diminished belief about its ability to influence outcomes^5^. Taken together, our results therefore suggest that MDD is associated with dysfunctions in both learning and decision-making.

Neuroticism is associated with a vulnerability to many common psychiatric disorders including depression^30,31^. Stress reactivity is thought to be a core aspect of neuroticism, with individuals scoring highly on neuroticism showing greater sensitivity to aversive (stressful) events^31^. A large population based study concluded that neuroticism increases vulnerability to depression because of increased sensitivity to stressful life events^32^. In addition to group differences discussed above, we were interested in exploring possible relationships between participants’ fitted model parameter values and questionnaire scores. Within control participants, across two different versions of the task, we recorded a negative relationship between self-reported neuroticism and a model parameter capturing a subject’s ability to use internal value estimates, meaning higher neuroticism scores were associated with a more variable decision process. Taking this exploratory analysis further, we found that this association also existed across healthy and MDD groups. However, we could not reliably distinguish this from a group-level effect, and future work is needed to address this.

In conclusion, our results demonstrate impairments in MDD in a probabilistic reward-learning task during both learning and decision-making phases of the experiment. Patients, naturally scoring higher on neuroticism than controls, had a decreased memory of previous rewards and were less able use internally estimated values to guide decision-making in our task.

## Electronic supplementary material

Supplementary information
