# Supplementary material for: Major Depression Impairs the Use of Reward Values for Decision-Making
Source: Sci Rep. 2018 Sep 14;8:13798. doi: 10.1038/s41598-018-31730-w (PMC6138642; doi:10.1038/s41598-018-31730-w)
Supplement: Supplementary file 1 — Supplementary information [file 41598_2018_31730_MOESM1_ESM.pdf]

Supplement

Major depression impairs the use of reward values for decision-making

S. Rupprechter, A. Stankevicius, Q. J. M. Huys, J. D. Steele, P. Seriès

Experiment Details

First, personality and clinical questionnaires were filled out and an interview was conducted, which lasted approximately one hour. This was followed by a training session lasting between 10 and 20 minutes. Final 15 minutes of preparation included subjects changing and a safety check by the NHS personnel. Scanning lasted approximately 50 minutes and the experiment ended with a 5 minutes debriefing session and monetary reimbursement. Every participant was paid £20. Their scores were converted into a percentage and rounded up. Performance-dependent bonus was defined as that percentage number divided by ten, so for example if they correctly responded in 66% of trials, they would receive an additional £7.

The 60 trials were divided into 4 periods, which again were split into three blocks each. After each period (every 15 minutes), there was a brief rest period. In each block, participants observed five different fractals exactly four times and made 5 decisions. Fractals were presented for 3 to 4 seconds and outcomes for 2.5 to 3.5 seconds. Decisions had to be made within 5 seconds. Null events (blank screens without interaction) and decisions (responding to a simple response prompt) were sometimes displayed (between 1.25 and 7.5 seconds) to obtain a baseline of brain activity.

Other modifications from the original task of Stankevicius et al. (6) included the display of the reward as a pound symbol instead of a treasure chest and simplified instructions that were more accessible to people suffering from depressive symptoms.

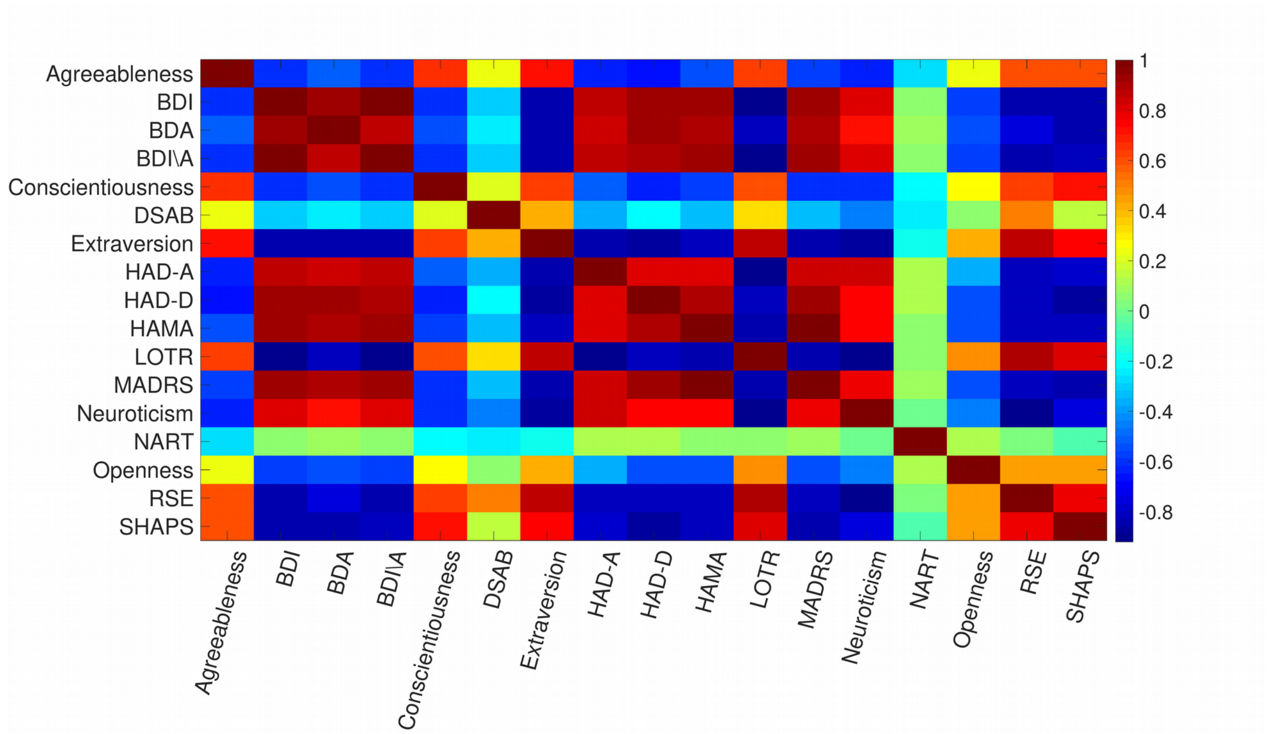

Figure S1. Correlation matrix of questionnaire scores of the fMRI dataset. As expected, questionnaire scores are often correlated with on another. See the section about additional analysis details below for more information about correlations between beta, neuroticism and other questionnaire scores.

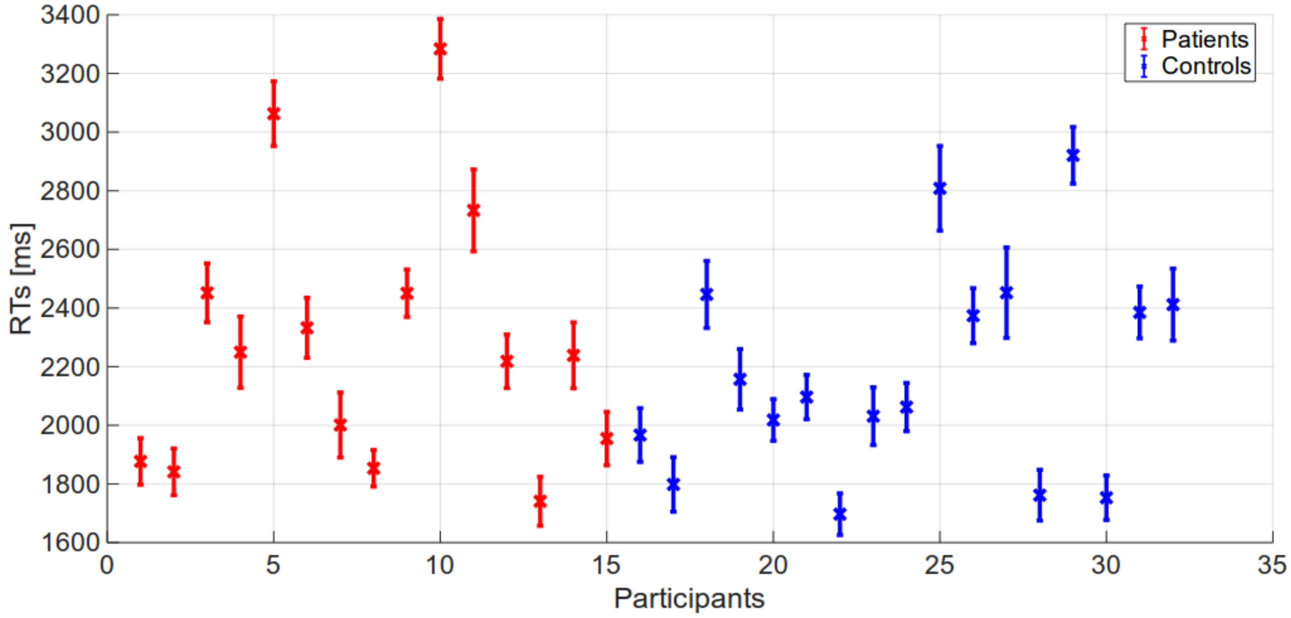

Figure S2. Average reaction times of participants in the fMRI dataset. Error bars represent standard errors. Mean response times were not significantly different between groups (Welch's t-test; t(26.6) = 0.692, p = .495).

Questionnaire	Patients	Controls	Score Range	p-value
fMRI dataset				
BDI	24.7 ± 13.1	4.2 ± 5.6	0 – 63	< 0.001
BDA	4.8 ± 2.9	0.5 ± 0.9	0 – 12	< 0.001
BDI\A	19.9 ± 10.8	3.7 ± 4.8	0 – 51	< 0.001
DSAB	15.0 ± 4.0	18.1 ± 2.7	0 – 24	0.013
HAD-A	12.3 ± 5.2	4.2 ± 2.3	0 – 21	< 0.001
HAD-D	8.6 ± 4.8	1.4 ± 2.1	0 – 21	< 0.001
HAMA	17.3 ± 7.0	1.4 ± 2.6	0 – 56	< 0.001
LOT-R	9.1 ± 5.5	18.4 ± 3.1	0 – 24	< 0.001
MADRS	17.7 ± 6.6	1.4 ± 2.6	0 – 60	< 0.001
NART	46.8 ± 4.2	46.6 ± 3.2	0 – 50	0.873
RSE	13.5 ± 6.9	24.5 ± 4.9	0 – 30	< 0.001
SHAPS	37.8 ± 8.5	49.6 ± 6.0	14 – 56	< 0.001
Agreeableness	39.5 ± 7.2	46.7 ± 5.9	12 – 60	0.004
Conscientiousness	36.3 ± 10.5	45.1 ± 7.0	12 – 60	0.008
Extraversion	30.5 ± 8.1	44.5 ± 5.2	12 – 60	< 0.001
Neuroticism	46.3 ± 7.1	29.8 ± 8.0	12 – 60	< 0.001
Openness	41.1 ± 5.1	46.5 ± 4.4	12 – 60	0.003
Pilot dataset				
BDI	27.7	10.1 ± 12.2	0 – 63	-
DSAB	13.7	14.8 ± 3.9	0 – 24	-
HAD-A	9.0	11.1 ± 3.5	0 – 21	-
HAD-D	7.3	8.4 ± 1.5	0 – 21	-
HAMA	18.0	5.1 ± 7.1	0 – 56	-
LOT-R	9.3	14.5 ± 5.5	0 – 24	-
MADRS	18.0	5.1 ± 7.1	0 – 60	-
NART	45.3	44.0 ± 11.3	0 – 50	-
RSE	18.7	9.5 ± 6.6	0 – 30	-
SHAPS	7.7	7.0 ± 1.1	14 – 56	-
Agreeableness	43.0	45.0 ± 5.8	12 – 60	-
Conscientiousness	32.0	43.3 ± 8.2	12 – 60	-
Extraversion	27.7	41.6 ± 6.9	12 – 60	-
Neuroticism	50.7	34.4 ± 11.5	12 – 60	-
Openness	46.7	46.2 ± 6.3	12 – 60	-

Table S1. Summary of questionnaire scores of participant groups and p-values for Welch's t-tests (fMRI dataset). Due the small number of patients included in the Pilot dataset, we did not calculate standard deviations for them and did not perform t-tests. BDI = Beck Depression Inventory; DSAB = Digit Score Part B; HAD = Hospital Anxiety and Depression Scale; HAMA = Hamilton Anxiety Rating Scale; LOT-R = Life Orientation Test – Revised; MADRS = Montgomery-Åsberg Depression Rating Scale; NART = National Adult Reading Test; RSE = Rosenberg Self-Esteem Scale; SHAPS = Snaith-Hamilton Pleasure Scale; Scores displayed as mean ± std.

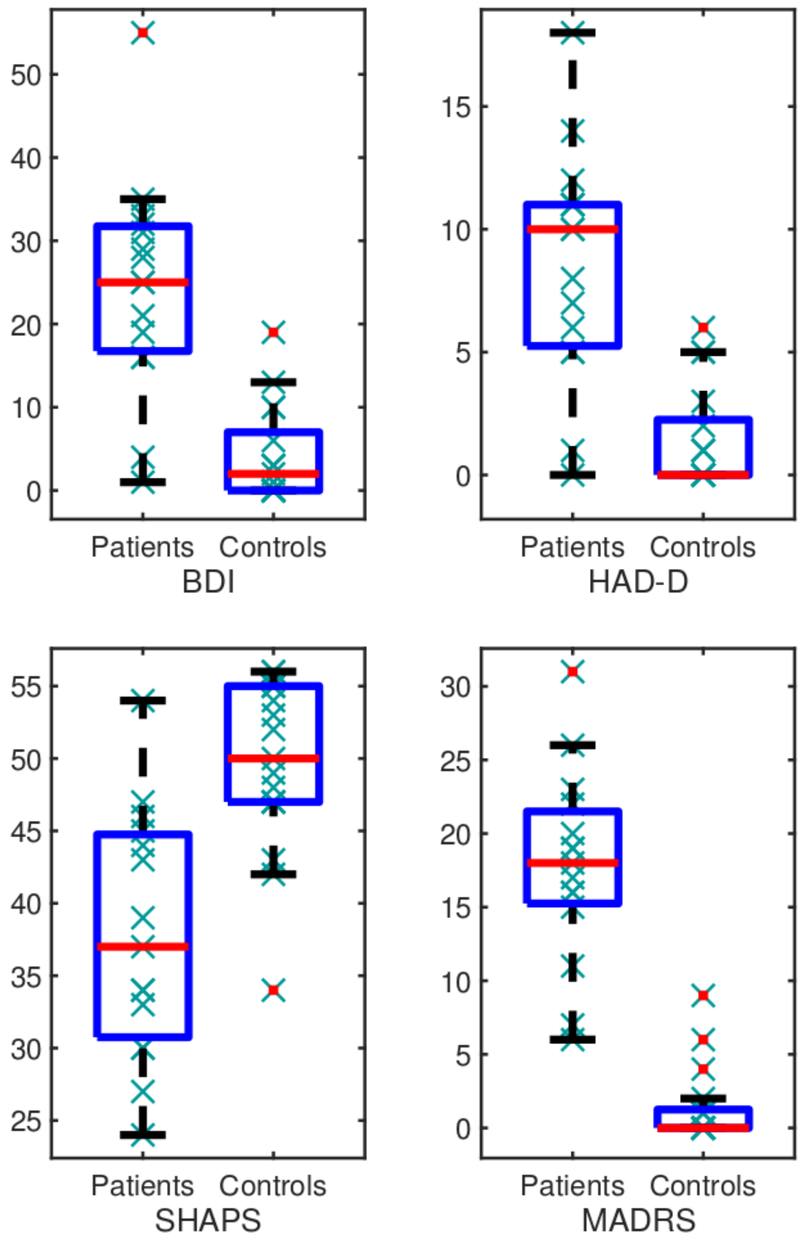

Figure S3. Boxplots of four questionnaires assessing depressive symptom severity (fMRI dataset). Patients had significantly higher scores than controls on the three scales measuring depressive severity (BDI, HAD-D, MADRS) and significantly lower scores on SHAPS, which measures pleasure (see Table S1).

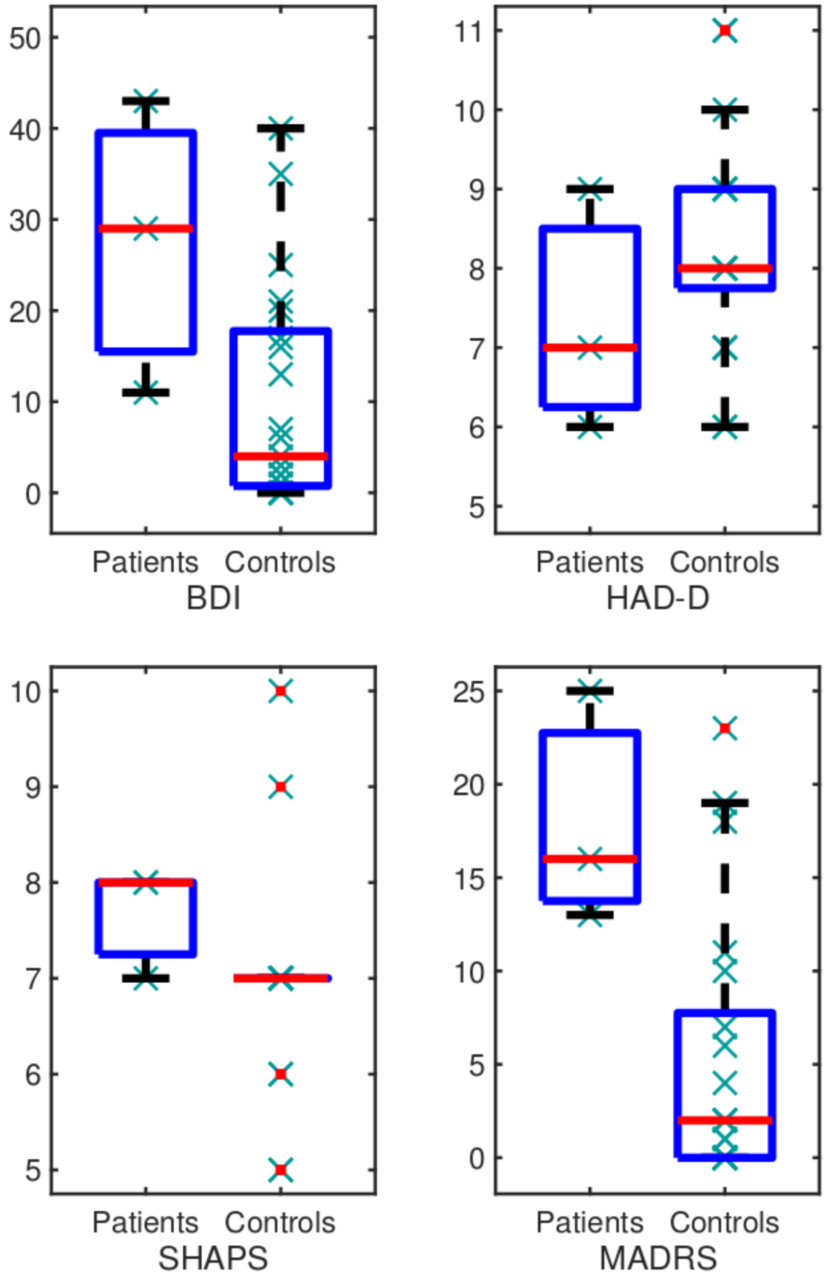

Figure S4. Boxplots of four questionnaires assessing depressive symptom severity (Pilot dataset). Note that the Snaith-Hamilton Pleasure Scale (SHAPS) was scored differently here: Each answer was scored either as zero (for two of the four possible answer options) or one (two alternative options), instead of scoring it one through four.

Additional Analyses Details

For our winning model we also tested whether our groups were better described using a shared population prior or separate priors for each group. Our data (fMRI dataset) was best described using a single population prior (ΔiBIC = 13.5).

Estimated model parameters were compared between groups using a Wilcoxon rank sum test which does not require an assumption of normality. (We used MATLAB's jbtest and lillietest to test for normality. In both cases the tests rejected the null hypothesis that the data comes from a normal distribution for the memory parameter for one of the groups, but not for the beta parameter. Using Welch's t-test instead gave us an almost identical result in terms of p value for the beta parameter.) Pearson correlation analysis across groups was performed, for which we reported classical p values. Below, we additionally used a Bayesian hypothesis test (1). A Bayes factor (BF10) larger than 3 indicates substantial evidence in favour of the alternative hypothesis (presence of a correlation). Classical p values may overestimate the evidence against the null hypothesis (2), but are also reported. All analyses were performed in MATLAB (R) R2017a (The MathWorks, Inc., Natick, MA).

Additional correlations with beta

Neuroticism was significantly negatively correlated with the inverse temperature parameter (r = -0.491, p = .004) across groups in the fMRI dataset (Figure S5). Because it is possible that this correlation is a function of group differences, we performed additional analyses taking into account group and also included our Pilot dataset after separately fitting the Leaky model. After controlling for group in the fMRI dataset, there was a non-significant but trending negative relationship between beta and neuroticism (r=-0.301, p=.100). This is unsurprising because the majority of our controls scored low in neuroticism, while most of our patients scored high. After controlling for group in the Pilot dataset, there was a significant negative correlation between beta and neuroticism (r=-0.433, p=0.039). Note that this dataset includes several control participants who scored high on neuroticism. In the pooled data, we again found a significant negative relationship between beta and neuroticism (t=-2.986, p=.004) after controlling for group and dataset version. Similarly, there was a significant negative relationship between beta and neuroticism (t(35)=-2.679, p=.011) combining only the control participants of both datasets, controlling for dataset version (reported in the main text). There was no significant correlation within the combined patients after correcting for dataset version (t(15)=-1.082, p=.297).

In the fMRI dataset (but not the Pilot dataset) there were also (weaker) positive correlations between β and extraversion ($r = 0.423$, BF10 = 2.46, $p = .016$), RSE ($r = 0.410$, BF10 = 2.01, $p = .020$), and LOT-R ($r = 0.382$, BF10 = 1.38, $p = .031$) scores, with this being at an “anecdotal” level of evidence (BF10 < 3). Since these scores were negatively correlated with neuroticism (Figure S1), we performed additional analyses to check whether neuroticism was the driving factor in these correlations and concluded that it was indeed so:

We fitted an additional general linear regression model (using MATLAB’s fitglm) including coefficients for neuroticism, extraversion, RSE, LOT-R, group membership and dataset version to the combined dataset. None of the coefficients were significant, but the p-value for neuroticism shows a trend ($t=-1.826$, $p=.074$), while all other coefficients were non-significant ($p > 0.6$), indicating that neuroticism should be the variable of interest (Table S2).

Model 1: `Beta ~ 1 + Neuroticism + Extraversion + RSE + LOTR + Group + Dataset`

We then created a linear regression model by stepwise regression (stepwiseglm), starting from the above Model 1, using the differences in the deviances of models as the criterion. Predictors where removed (stepwise) if the deviance was greater than 0.05 and only neuroticism remained in the final model ($t=-3.286$, $p=.002$):

Model 2: `Beta ~ 1 + Neuroticism`

This shows that indeed neuroticism is the variable of interest and was the driving factor for the correlations between the other variables and beta in the fMRI dataset and suggests a possible link between participants' neuroticism and their difficulty in making decisions based on their internal value estimations across groups.

**Coefficient**	**t**	**p**
Neuroticism	-1.826	**0.074**
Extraversion	-0.321	0.749
RSE	-0.290	0.772
LOT-R	0.367	0.715
Group	-0.437	0.664
Dataset Version	-0.510	0.612

Table S2. Results of t-tests on individual coefficients of Model 1.

Optimism and previous results

The original Life Orientation Test (LOT) was revised (3) to the current form (LOT-R) after criticism that LOT scores could not be distinguished from neuroticism scores and correlations with optimism disappeared when controlled for neuroticism (4). However, it has been shown that even these LOT-R scores are not independent from neuroticism scores (5). After re-analysing the published data from Stankevicius et al. (6) using the methods described here (see Model Fitting Procedure), we were not only able to confirm that LOT-R scores corresponded to a prior belief about rewards ($r = .524$, $p < 0.001$), but also found that neuroticism was similarly related to a (negative) prior belief ($r = -.327$, $p = 0.019$).

In the present fMRI data, the prior mean did not significantly correlate with LOT-R ($r=-0.255$, $p=.159$) nor with neuroticism ($r=0.322$, $p=.072$), nor with the first principal component of our questionnaires measuring depression severity ($r=0.115$, $p=.532$), nor with any of the individual depression questionnaire scores (BDI, HAD-D, MADRS, SHAPS), suggesting that neither optimism, nor neuroticism, nor depression biased participants towards choosing a certain option.

We do not know for certain why we were unable to directly replicate previous results, but we have several hypotheses and are planning to address this in future work. Overall, we think that we introduced too many important changes from the original experiment and so the tasks are not directly comparable any more: In our Pilot and fMRI experiments, trials lasted a lot longer than in the published task (several seconds instead of fractions of a second). This was necessary to be able to capture the BOLD response during scanning, but initially made the task too easy. We tried to compensate for that by reducing the space of possible differences between the probability of reward associated with the two targets (-30% to 30% instead of -100% to 100%), which introduced much more uncertainty in the decisions. It is likely that the scanner induced additional uncertainty and pressure and so overall the task was very hard.

There were other differences as well: Each fractal was observed a variable number of times in the published data, while in the novel tasks each fractal was observed exactly four times. While participants only performed a short trial version of the task in the original version, here we trained our subjects extensively (between 10 and 20 minutes) before the main experiment. In addition, (a) there might be trait-cohorts interactions, (b) we might not have enough participants to reliably find the effect, (c) the saliency of the rewards might have been different (empty or full treasure chest versus a pound symbol or empty screen), and (d) the performance-related monetary reward the participants received after the experiments was different and likely led to differences in motivations.

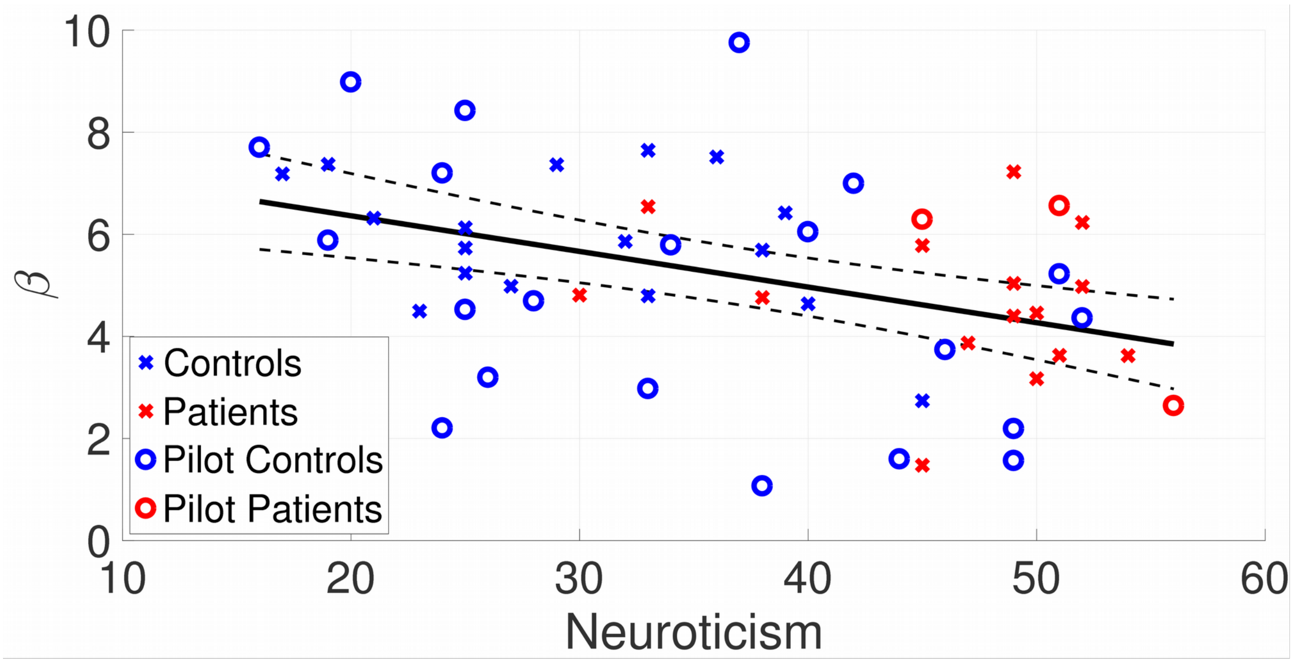

Figure S5. A scatter plot of neuroticism and β, a parameter in our best fitting Leaky model capturing participants' ability to follow internal value estimations, and their correlation across patients and control subjects of both datasets (shown without controlling for group or dataset version: r=-0.408, p=.002; regression line with 95% confidence interval; after controlling for both group and dataset version: t=-2.986, p=.004).

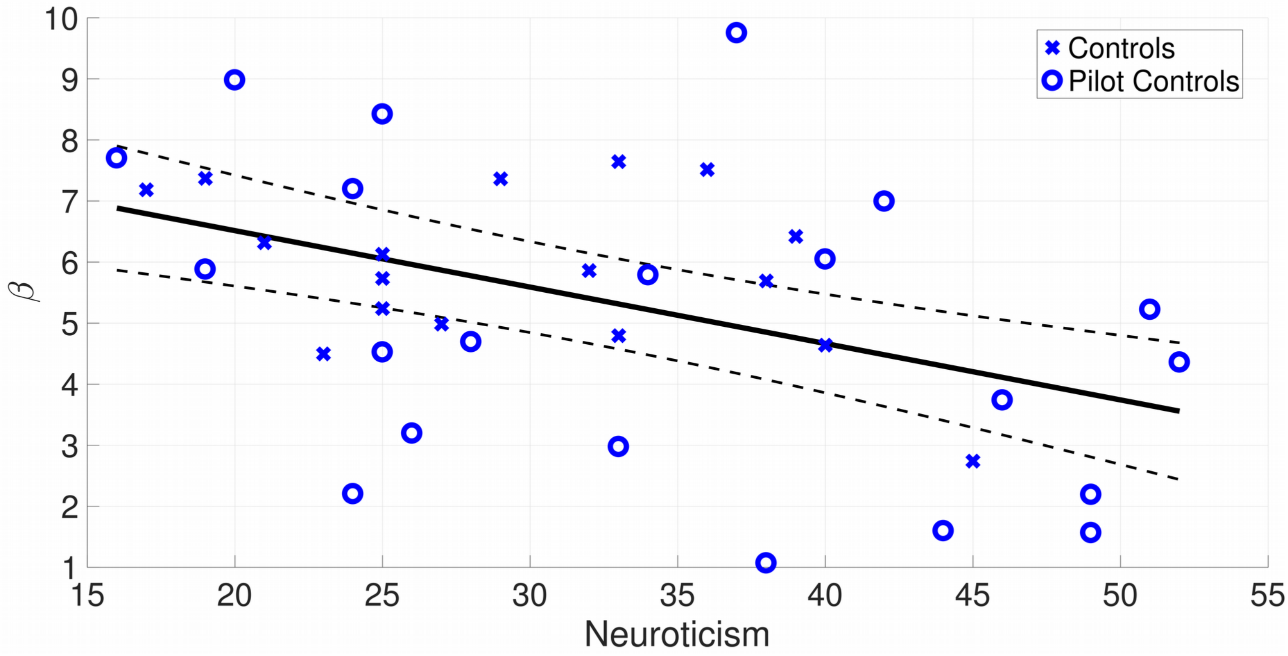

Figure S6. A scatter plot of neuroticism and β, a parameter in our best fitting Leaky model capturing participants' ability to follow internal value estimations, and their correlation across control subjects of both datasets (without controlling for dataset version; regression line with 95% confidence interval).

Bayesian model

The Bayesian model was introduced in detail by Stankevicius et al. (6). We briefly describe it here for completeness:

At each decision point, participants are assumed to know how often the fractal *i* used in the decision was shown (N_i) and how often it was followed by reward (n_i). It is further assumed that subjects behave as Bayesian observers and use Bayes rule to compute a posterior, from which they extract the mean to make decisions. The prior is modelled as a Beta distribution, which is conjugate to the binomial distribution. It can be shown that the posterior mean takes on the following form:

$$m_i = \frac{n_i + \alpha}{N_i + \alpha + \beta}$$

where alpha and beta control the shape of the Beta distribution. The mean is then plugged into a softmax function to obtain the probability of choosing fractal *i* as in the other models:

$$\sigma(\gamma(m_i - \phi_i))$$

Reward Sensitivity

In previous work (12), the inverse temperature parameter has also been interpreted as reward sensitivity and it has been argued that in some cases it can be substituted exactly for a reward sensitivity parameter (9) in a RL model. This however only holds when all options in the softmax are estimated from observed rewards, which is not the case here, as we show in an example:

In our model, on the first timestep we will have $V^{(1)} = \rho r^1$. After the second timestep we will have $V^{(2)} = A V^{(1)} + \rho r^{(2)}$ which we can rewrite as $V^{(2)} = \rho \left(A r^{(1)} + r^{(2)} \right)$. In general, after each step we will have a V value that is a combination of A and r scaled by ρ . Within the softmax function there is a subtraction term $x - y$ which is multiplied by β . This can be rewritten as $\beta x - \beta y$, which means that the β parameter scales both x and y , just as ρ would scale each of the variables *if they are both estimated on a trial-by-trial basis*. However, if one of these values is instead fixed (as is the case for our explicit probability), ρ will only scale one of the variables, while β will still scale both of them, which makes the parameters distinguishable.

Our beta parameter should therefore not be interpreted as being equivalent to a reward sensitivity parameter. In our model, a high inverse temperature likely indicates that participants were able to perform better in the highly uncertain environment and put more trust in their own estimations. Lower beta values indicate that participants put less trust in their estimations and chose more randomly. It is perfectly possible (and given previous research it is indeed likely) that patients were less reward sensitive than controls, and some of this difference may be captured by the beta parameter. It is, for example, possible that patients put less trust in their estimations because they were less sensitive to the rewards in the first place, but this is not explicitly modelled here. To reliably distinguish beta from reward sensitivity, we would need an additional reward sensitivity parameter, which we included in one of our models (Leaky-rho). However, model comparison did not reveal this to be the most parsimonious model and so we did not pursue this further.

Model Simulations

We provide additional mesh plots where we simulated data using the model while we systematically varied both parameters (Figure S7). The plots show that variations in the two parameters lead to different effects: While the number of correct responses alone can not be used to distinguish

between the parameters, the effects are very different when separately looking at trials on which a fractal response was correct and trials on which an explicit probability response was correct.

There is a clear positive correlation between the beta value and the (average) number of correct responses on trials on which the explicit option was correct. Variations in the memory parameter have little effect in that case. However, the memory parameter is important on trials on which the fractal was the correct choice and higher values lead to more correct choices. The beta parameter modulates this relationship between memory and number of correct choices. High beta values results in a large effect of memory on the number of correct responses, while low beta values flatten out this effect.

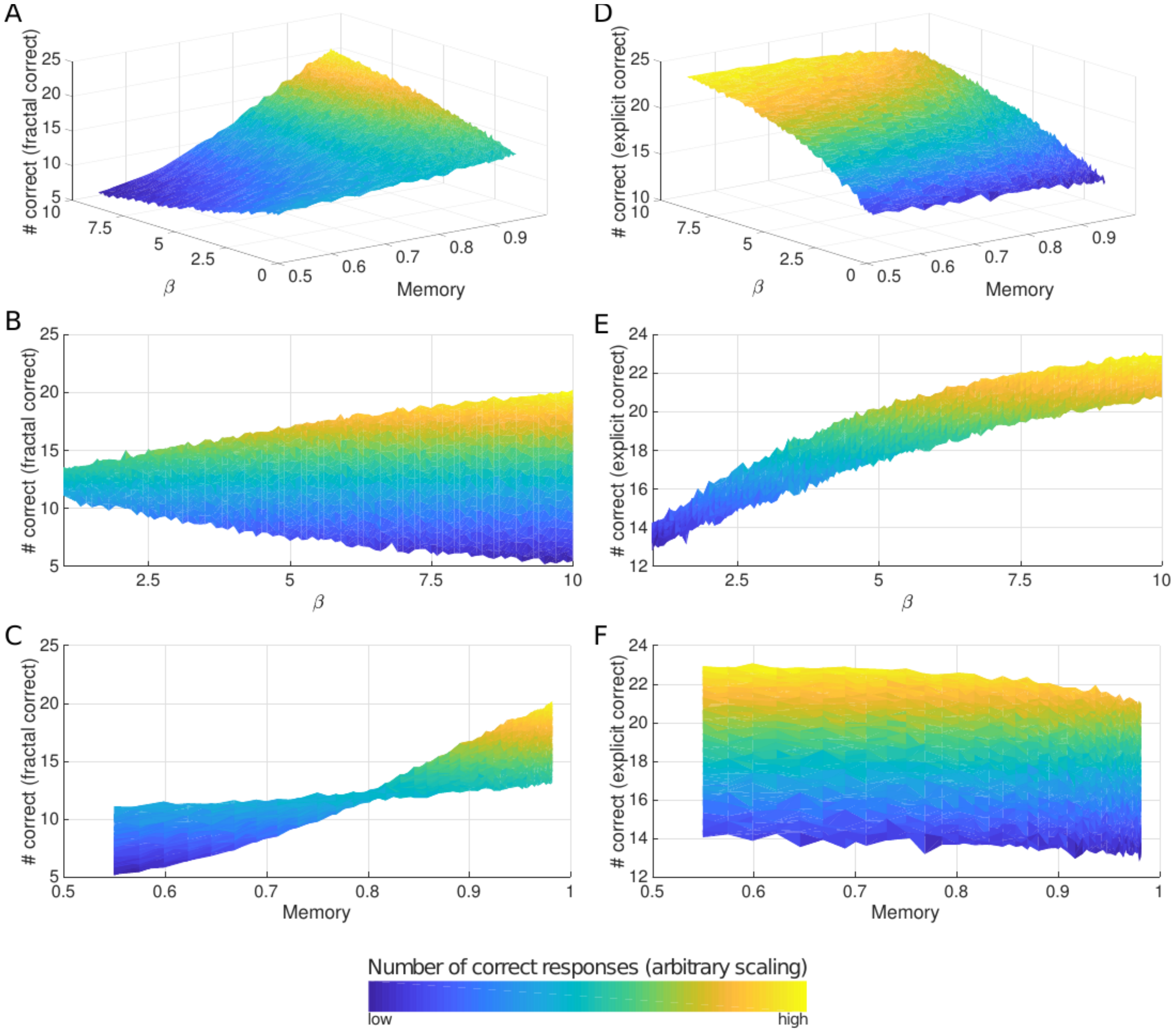

Figure S7. Simulations of our model using different parameter values for memory (discount parameter A) and β. The number of correct responses averaged over 100 simulations is shown. The left column (A-C) shows the number of correct choices on trials for which 'fractal' was the correct response as a function of the two parameters from different viewpoints of the grid. The right column (D-F) shows the same for the number of correct choices on trials for which the explicit probability was the correct response.

Model Fitting Procedure

We used model fitting and comparison procedures that have been used previously by Huys et al. (7, 8, 9). For completeness, we will describe them here in detail.

The goal of the model fitting procedure is to find estimates of the parameter vector θ_i for each participant *i*. This can be done by maximizing the probability that the observed choice data C_i came from the distribution governed by this θ:

$$\theta_i^{ML} = \underset{\theta}{\operatorname{argmax}}\, p(\mathcal{C}_i|\theta).$$

Repeating this procedure for each participant separately without any constraints can however lead to poor estimates and ignores the fact that we would expect parameters of different individuals to be comparable (e.g. to be of the same order of magnitude). One simple way to deal with this would be to enforce hard constraints on the parameter estimates, but a more principled way is to use a maximum a posteriori estimate and add a prior with information about the likely range of parameter values (10):

$$\theta_i^{MAP} = \underset{\theta}{\operatorname{argmax}}\, p(\mathcal{C}_i|\theta)\; p(\theta).$$

One option for such a prior is to estimate it from the data. Making the random effects assumption that parameters of individuals are samples from an overall group distribution and that this distribution is a Normal distribution with mean μ and variance Σ, we can use Expectation-Maximisation to simultaneously estimate group and individual parameters (7, 8, 9).

In the E-step (kth iteration) a Laplace approximation (mean mu and variance V) is used to estimate the parameters of individuals:

$$\begin{aligned} p(\theta|\mathcal{C}_i) &\approx \mathcal{N}(\boldsymbol{m}_i^{(k)}, \boldsymbol{V_i}^{(k)}) \\ \boldsymbol{m}_i^{(k)} &= \underset{\boldsymbol{m}}{\operatorname{argmax}}\; p(\mathcal{C}_i|\boldsymbol{m})\; p(\theta_i|\boldsymbol{\mu}^{(k-1)}, \boldsymbol{\Sigma}^{(k-1)}), \end{aligned}$$

In the M-step the population parameters are updated, taking into account the uncertainty of parameter estimations of individuals (weighted mean vector and covariance matrix):

$$\begin{aligned} \boldsymbol{\mu}^{(k)} &= \Big(\sum_{i=1}^{N} \boldsymbol{V_i}^{-1}\Big)^{-1} \Big(\sum_{i=1}^{N} \boldsymbol{V_i}^{-1}\boldsymbol{m}_i\Big) \\ \boldsymbol{\Sigma}^{(k)} &= \frac{1}{N-1}\sum_{i=1}^{N} (\boldsymbol{m}_i - \boldsymbol{\mu})(\boldsymbol{m}_i - \boldsymbol{\mu})^{\mathsf{T}} + \boldsymbol{V_i} \end{aligned}$$

To enforce constraints, parameters were transformed through non-linear functions with support on the real line. To avoid falling into local minima, multiple random initialisations were used. The procedure proved to be quite stable over multiple runs, repeatedly estimating very similar parameters.

Model Comparison Procedure

Having fitted our models, we want to find out which model has the highest probability of being the correct model given our data (11). This means that for some model M we are interested in finding its posterior probability

$$P(\mathcal{M}|\mathcal{C}) = \frac{P(\mathcal{C}|\mathcal{M})\ P(\mathcal{M})}{P(\mathcal{C})}.$$

The probability of the choice data P(C) will be the same under all models and since we have no prior preference for any of the models, P(M) will also be equal for all models, which means that when we take the ratio of posterior model probabilities they will both cancel out. The model evidence remains and can be rewritten as

$$P(\mathcal{C}|\mathcal{M}) = \int d\boldsymbol{\theta}\ P(\mathcal{C}|\boldsymbol{\theta}, \mathcal{M})\ P(\boldsymbol{\theta}|\mathcal{M}),$$

which we then approximate (7, 8, 9) with

$$\log(P(\mathcal{C}|\mathcal{M})) \approx \log(P(\mathcal{C}|\hat{\theta}, \mathcal{M})) - \frac{1}{2}|\mathcal{M}|\log(|\mathcal{C}|) = -\frac{1}{2}\text{iBIC},$$

where |M| is the number of fitted prior parameters, |C| is the overall number of choices and the difference in iBIC values of two models will be an approximation to the log Bayes Factor (9). The “i” in front of BIC stands for “integrated”, because to compute log(p(C|$\hat{\theta}$, M)) we integrate over parameters so that

$$\log(p(\mathcal{C}|\hat{\boldsymbol{\theta}}, \mathcal{M})) = \sum_{i=1}^{N}\log(P(\mathcal{C}_i|\hat{\boldsymbol{\theta}}, \mathcal{M})) = \sum_{i=1}^{N}\log\Big(\int d\boldsymbol{h}\ P(\mathcal{C}_i|\boldsymbol{h}, \mathcal{M})\ P(\boldsymbol{h}|\hat{\boldsymbol{\theta}}, \mathcal{M})\Big).$$

This can be approximated by sampling from our estimate prior and averaging over those samples (7, 8, 9):

$$\begin{aligned} \boldsymbol{h}^{(s)} &\sim p(\boldsymbol{h}|\hat{\boldsymbol{\theta}}, \mathcal{M}) \\ \log(p(\mathcal{C}|\hat{\boldsymbol{\theta}}, \mathcal{M})) &\approx \sum_{i=1}^{N}\log\Big(\frac{1}{S}\sum_{s=1}^{S}p(\mathcal{C}_i|\boldsymbol{h}^{(s)}, \mathcal{M})\Big) \end{aligned}$$

For our winning model we also tested whether our groups were better described using a shared population prior or separate priors for each group. For this, iBIC values of the fits of separate group

priors were added and compared to the iBIC value of a single population prior. Our data was best described using a single population prior (ΔiBIC = 13.5).

Simulations

It is important to check that models can be recovered given the available data and so we performed model recovery simulations. Models were simulated to produce the same amount of actually available data (32 subjects, 60 decisions) and then the generating model and an alternative model were fit to the data and standard model comparison was used to decide which model produced the better fit.

These simulations showed that Leaky and Leaky-ρ could not reliably be distinguished with the amount of data we have and Leaky was often selected as best model even when Leaky-ρ produced the data. Importantly, however, our second and third best-fitting reinforcement learning models could reliably be recovered from data they generated in most simulations as shown in Table S3.

**Bayesian**	**RL-basic**	**RL-learning**	**RL-unbiased**	**RL-learning-unbiased**
12/15	12/15	15/15	14/15	15/15

Table S3. Model recovery table showing how often each model was recovered from data they had generated (15 simulations) against the Leaky model. Leaky always had the lower iBIC value (15/15 against all models) when fitted to data it had generated.

To check whether the model fitting procedure is actually able to recover parameters given the amount of data available to us, we simulated 32 participants with 60 decisions each from known parameters. To get sensible parameter values, we randomly sampled from the estimated group prior. Errors were consistently lower for our procedure than standard maximum likelihood estimations and our procedure also avoids outliers sometimes produced by MLE by pushing estimations towards the group mean. Figures S8 and S9 show a first example of the recovery of memory A and inverse temperature β parameters for the model Leaky, while Figures S10 and S11 show the same for a second example and Figures S12 and S13 for a third example. Simulations of other models showed similar parameter recovery.

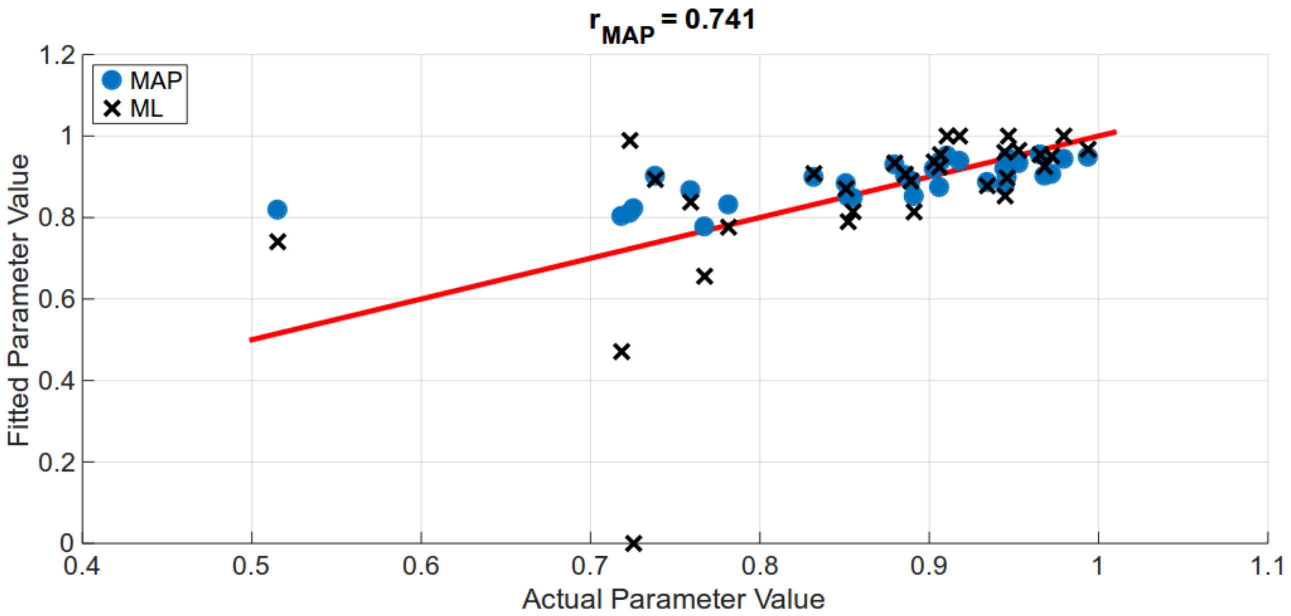

Figure S8. Example 1 (A): Parameter recovery simulation of the memory parameter A using a group prior (MAP) and simple maximum likelihood (ML). The red line shows the values with which the data was actually generated. Note how MAP estimation leads to improvements over ML estimation, in particular by eliminating the outlier lying on the zero boundary.

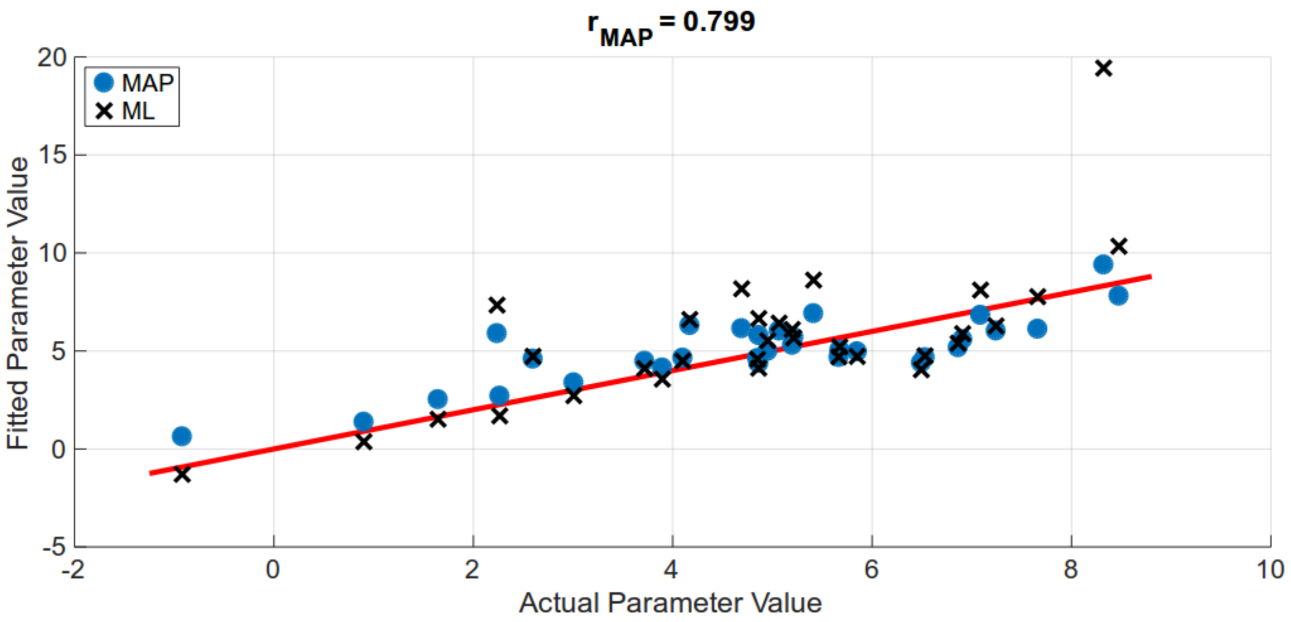

Figure S9. Example 1 (β): Parameter recovery simulation of the inverse temperature parameter β using a group prior (MAP) and simple maximum likelihood (ML). The red line shows the values with which the data was actually generated.

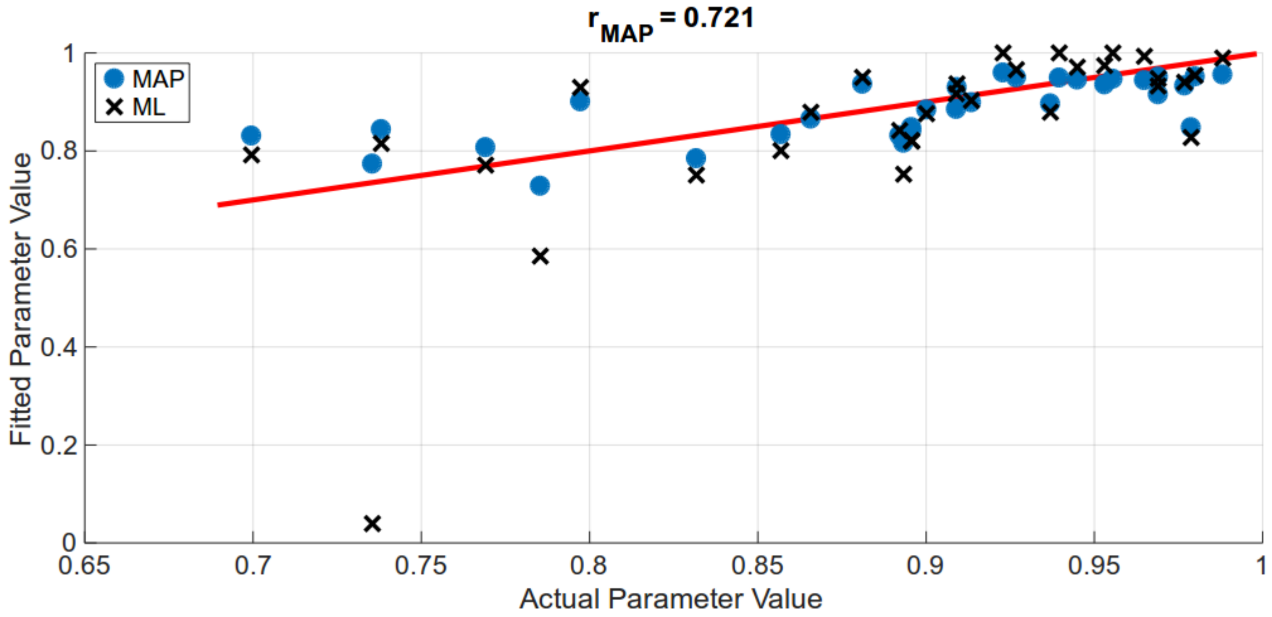

Figure S10. Example 2 (A): Parameter recovery simulation of the memory parameter A using a group prior (MAP) and simple maximum likelihood (ML). The red line shows the values with which the data was actually generated.

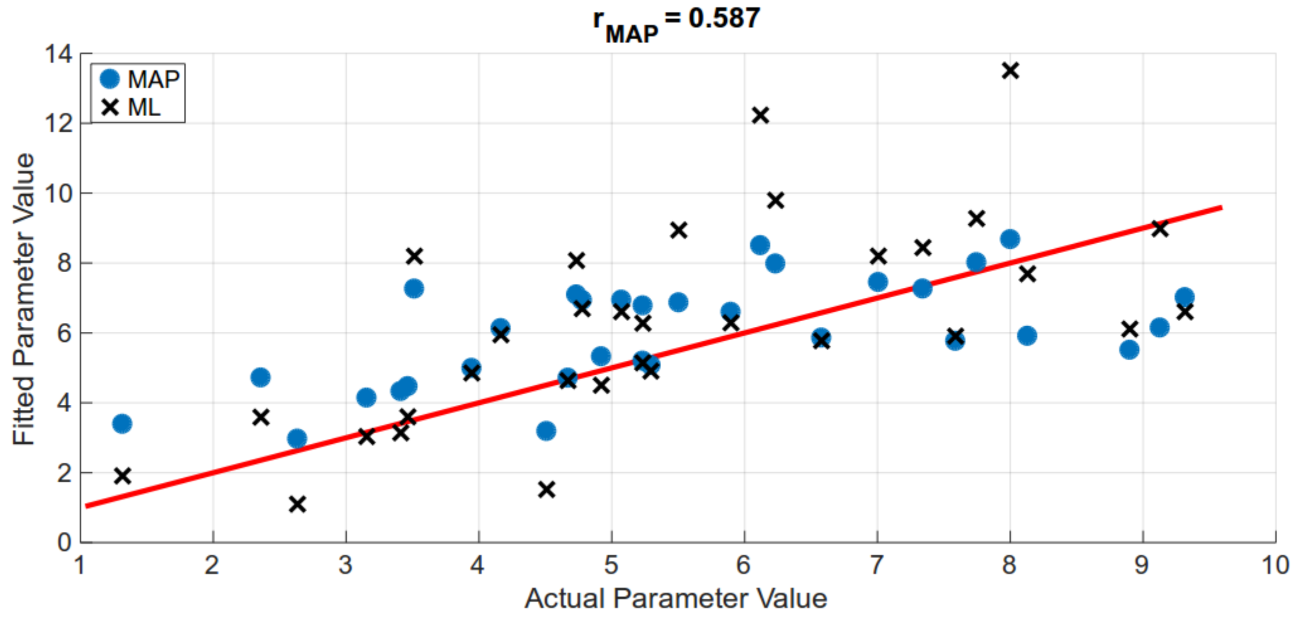

Figure S11. Example 2 (β): Parameter recovery simulation of the inverse temperature parameter β using a group prior (MAP) and simple maximum likelihood (ML). The red line shows the values with which the data was actually generated.

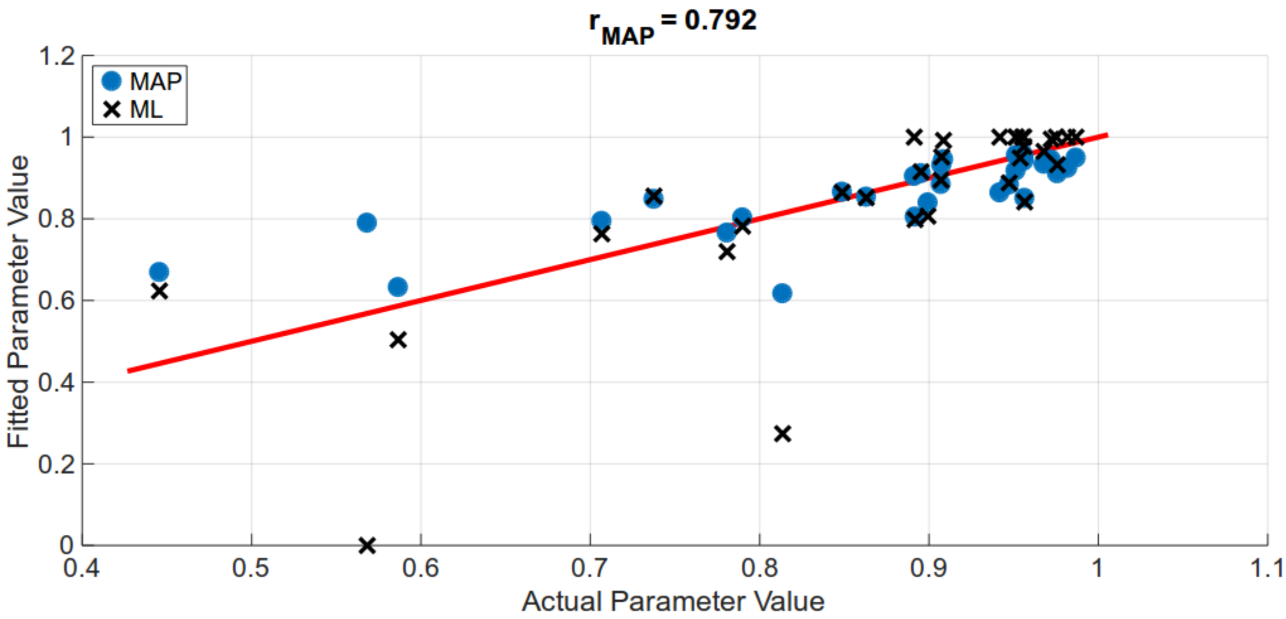

Figure S12. Example 3 (A): Parameter recovery simulation of the memory parameter A using a group prior (MAP) and simple maximum likelihood (ML). The red line shows the values with which the data was actually generated.

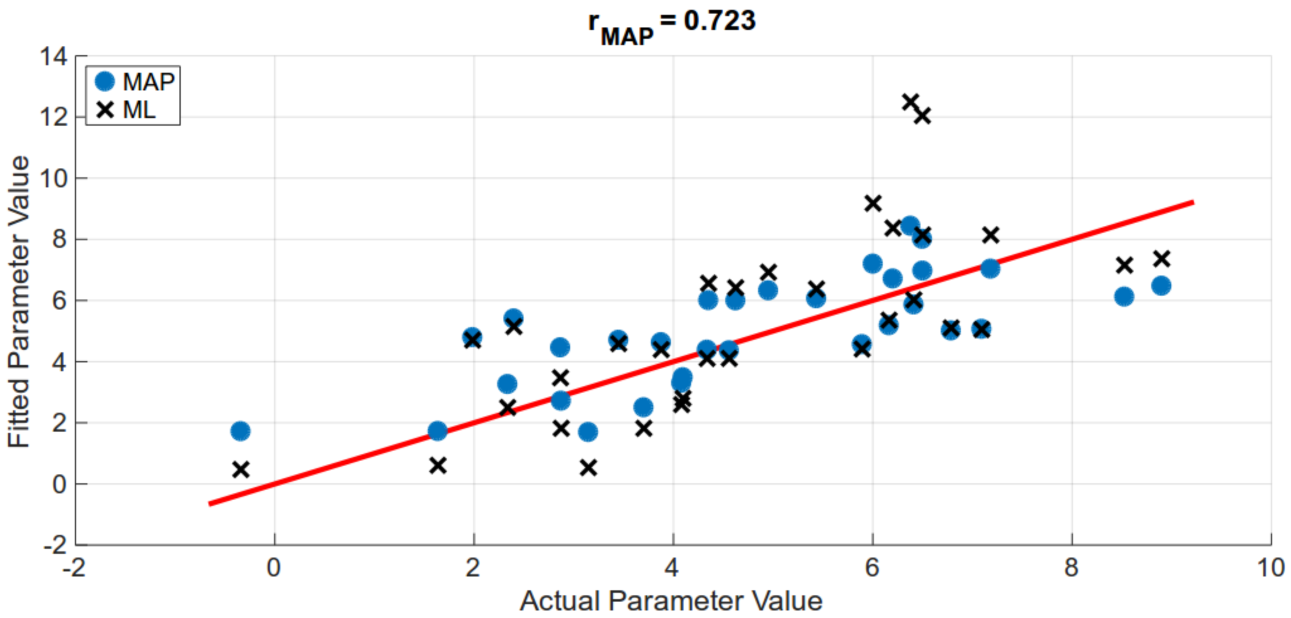

Figure S13. Example 3 (β): Parameter recovery simulation of the inverse temperature parameter β using a group prior (MAP) and simple maximum likelihood (ML). The red line shows the values with which the data was actually generated.

Parameter correlation

There was a trend suggesting a correlation between parameter estimates ($r = 0.349$, BF10 = 0.91, $p = .051$). We performed additional parameter recovery simulations (Figures S14, S15, S16) in which we systematically varied parameters to further convince ourselves that parameter correlations did not systematically influence the fitting of parameters. We simulated individual participants, with one of the values fixed to be the same for everybody (e.g. setting the memory $A=0.9$) while varying the other parameter across participants (e.g. have participants with betas in the range of 4 until 9). Most importantly, for the beta parameter we did not find that different realistic values of the memory parameter had a systematic influence on the quality of the parameter recovery (Figure S14).

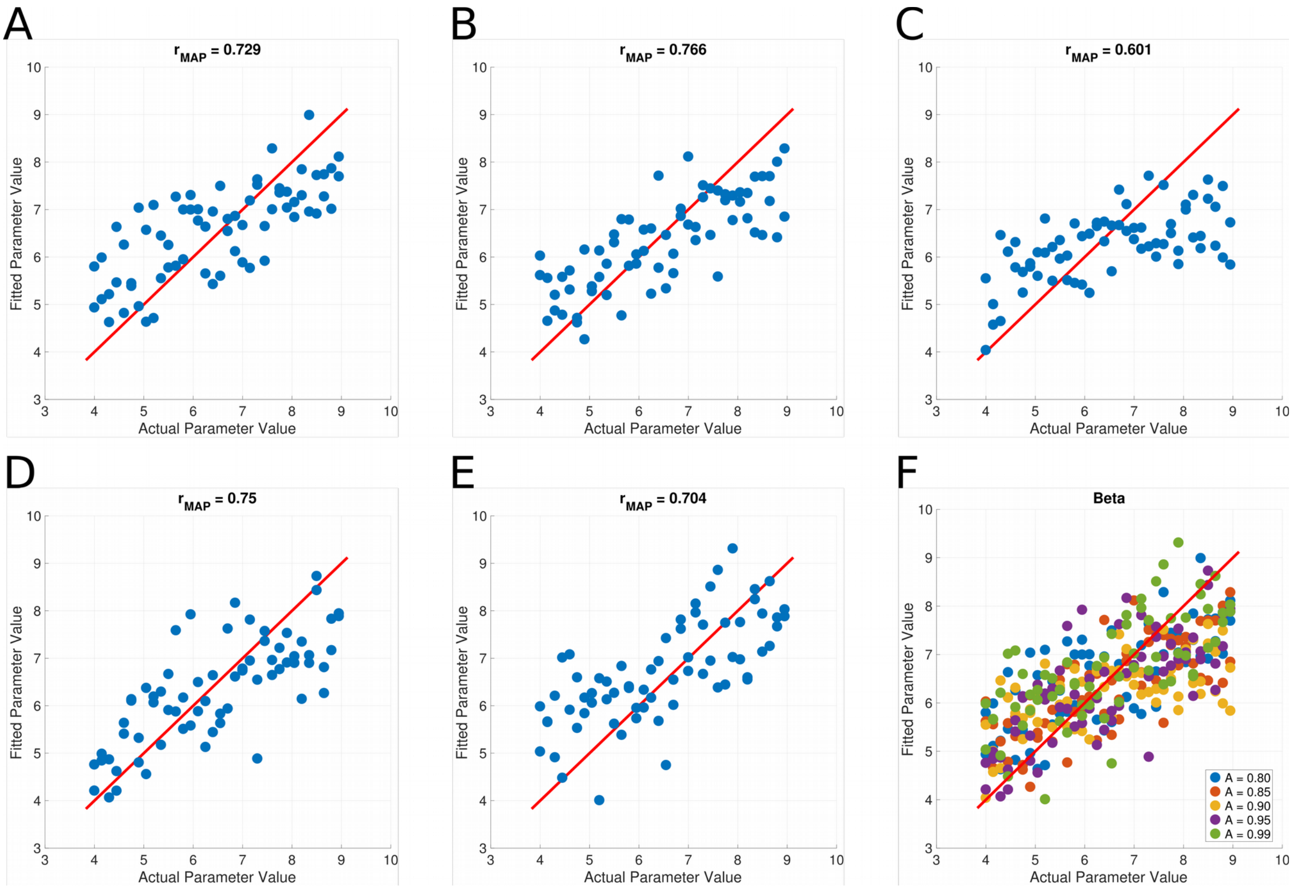

Figure S14. Recovery of the beta parameter, while fixing the memory parameter *A* to certain realistic values: (A) *A*=0.80, (B) *A*=0.85, (C) *A*=0.90, (D) *A*=0.95, (E) *A*=0.99. Subfigure (F) shows (A-E) combined. For each recovery plot, 68 participants were simulated, making 120 decisions. It can be seen that the parameter recovery of the beta parameter is not systematically influenced by the setting of the memory parameter.

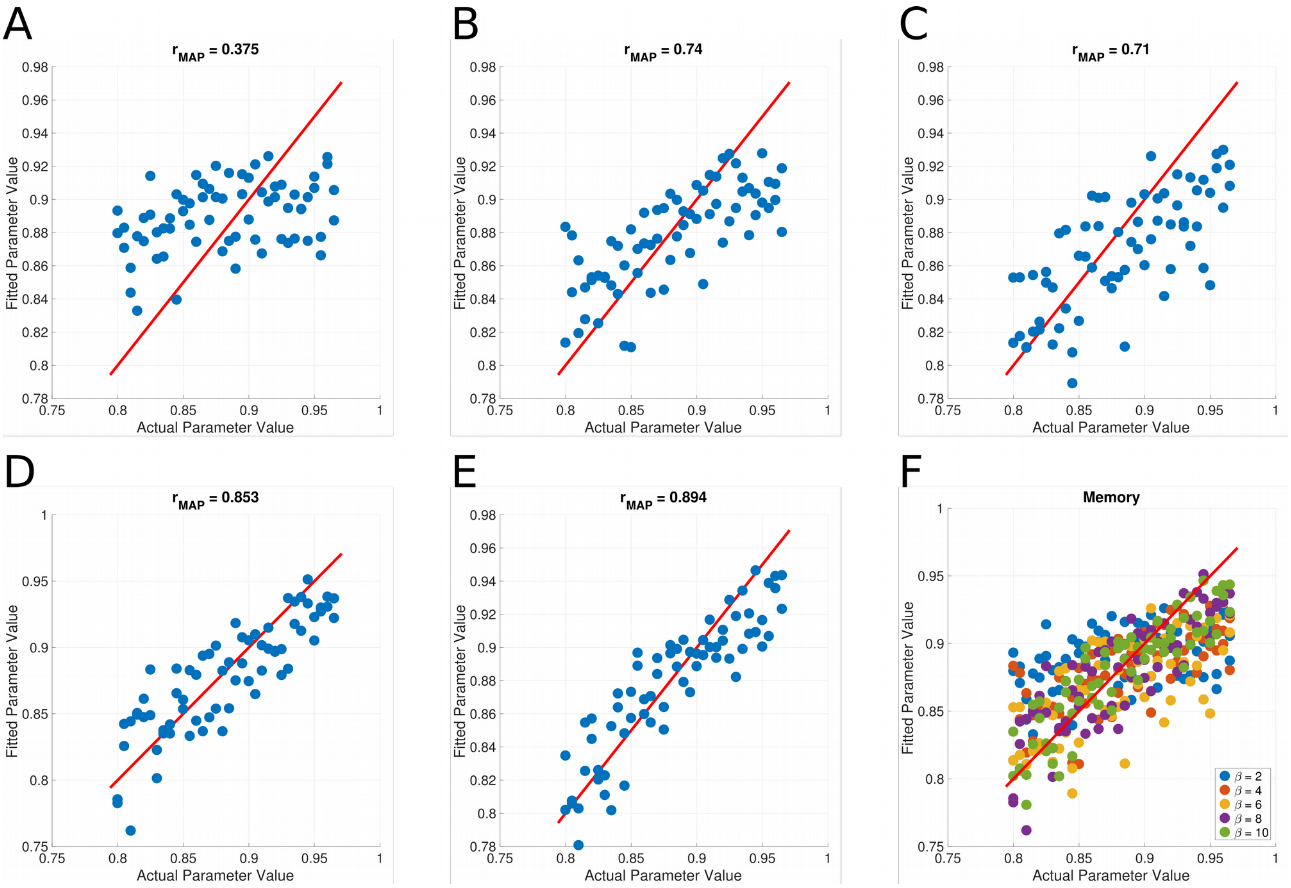

Figure S15. Recovery of the memory (*A*) parameter, while fixing the inverse temperature parameter β to certain values: (A) β=2, (B) β=4, (C) β=6, (D) β=8, (E) β=10. Subfigure (F) shows (A-E) combined. For each recovery plot, 68 participants were simulated, making 120 decisions. For very low (and probably mostly unrealistic) β values, the recovery of the memory parameter was noticeably worse than for all the other more realistic values of 4 and above.

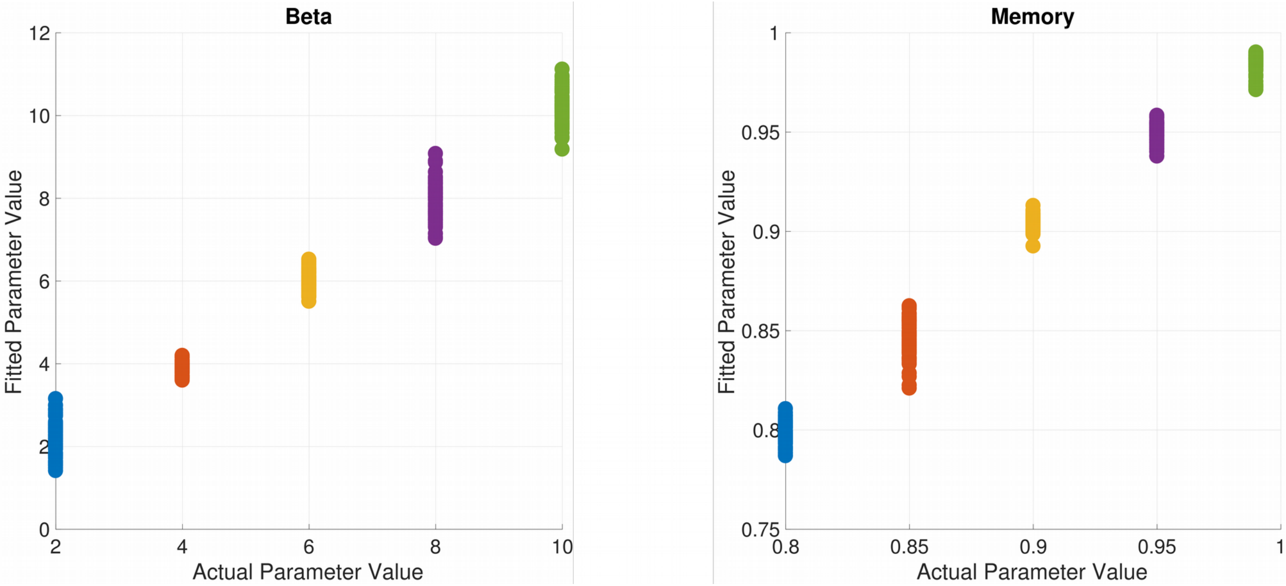

Figure S16. Recovery of the fixed parameters. (Left) Beta parameters were fixed at values 2, 4, 6, 8, and 10 and recovered well. The corresponding recoveries of the systematically varying memory parameters are shown in Figure S14. (Right) Memory parameters were fixed at values 0.80, 0.85, 0.90, 0.95, and 0.99 and recovered well. The corresponding recoveries of the systematically varying beta parameters are shown in Figure S15.

Supplementary References

[1] R. Wetzels and E.-J. Wagenmakers, “A default Bayesian hypothesis test for correlations and partial correlations,” Psychonomic bulletin & review, vol. 19, no. 6, pp. 1057–1064, 2012.

[2] J. O. Berger and T. Sellke, “Testing a point null hypothesis: The irreconcilability of p values and evidence,” Journal of the American statistical Association, vol. 82, no. 397, pp. 112–122, 1987.

[3] M. F. Scheier, C. S. Carver, and M. W. Bridges, “Distinguishing optimism from neuroticism (and trait anxiety, self-mastery, and self-esteem): a reevaluation of the life orientation test.,” Journal of personality and social psychology, vol. 67, no. 6, p. 1063, 1994.

[4] T. W. Smith, M. K. Pope, F. Rhodewalt, and J. L. Poulton, “Optimism, neuroticism, coping, and symptom reports: An alternative interpretation of the life orientation test.,” Journal of Personality and Social Psychology, vol. 56, no. 4, p. 640, 1989.

[5] D. K. Kennedy and B. M. Hughes, “The optimism-neuroticism question: An evaluation based on cardiovascular reactivity in female college students,” The psychological record, vol. 54, no. 3, p. 373, 2004.

[6] Stankevicius, A., Huys, Q. J., Kalra, A., and Seriès, P. (2014). Optimism as a prior belief about the probability of future reward. PLoS Computational Biology, 10(5):e1003605.

[7] Huys, Q. J., Cools, R., Gölzer, M., Friedel, E., Heinz, A., Dolan, R. J., and Dayan, P. (2011). Disentangling the roles of approach, activation and valence in instrumental and Pavlovian responding. PloS Computational Biology, 7(4):e1002028.

[8] Huys, Q. J., Eshel, N., O’Nions, E., Sheridan, L., Dayan, P., and Roiser, J. P. (2012). Bonsai trees in your head: how the pavlovian system sculpts goal-directed choices by pruning decision trees. PloS Computational Biology, 8(3):e1002410.

[9] Huys, Q. J., Pizzagalli, D. A., Bogdan, R., and Dayan, P. (2013). Mapping anhedonia onto reinforcement learning: a behavioural meta-analysis. Biology of mood & anxiety disorders, 3(1):12.

[10] Daw, N. D. (2011). Trial-by-trial data analysis using computational models. Decision making, affect, and learning: Attention and performance XXIII, 23:3–38.

[11] MacKay, D. J. (2003). Information theory, inference and learning algorithms. Cambridge University Press.

[12] Huys, Q. J., Vogelstein, J. T., Dayan, P. & Bottou, L. Psychiatry: Insights into depression through normative decision-making models. In NIPS, 729–736 (2008).
